# Metaphor Comprehension in Schizophrenic Patients

**DOI:** 10.3389/fpsyg.2018.00670

**Published:** 2018-05-09

**Authors:** Ileana Rossetti, Paolo Brambilla, Costanza Papagno

**Affiliations:** ^1^Dipartimento di Psicologia, Università di Milano-Bicocca, Milan, Italy; ^2^Scientific Institute IRCCS “E. Medea”, Bosisio Parini, Italy; ^3^Department of Pathophysiology and Transplantation, University of Milan, Milan, Italy; ^4^Center for Mind/Brain Sciences and Centro di Riabilitazione Neurocognitiva, University of Trento, Trento, Italy

**Keywords:** metaphor, schizophrenia, psychosis, figurative, concretism, first episode psychosis

## Abstract

People with schizophrenia often exhibit difficulties to comprehend figurative expressions, such as irony, proverbs, metaphors and idioms, with a general proneness to neglect the figurative meaning and to accept the more literal one. This inability is usually referred to as concretism and it constitutes a clinical manifestation of the broader language dysfunction called Formal Thought Disorder. The current review focuses on the neuropsychological and neuroanatomical underpinnings of schizophrenics’ misinterpretation of a subgroup of figurative expressions, i.e., metaphors. Metaphors are heterogeneous in nature, classifiable according to various criteria; for instance, metaphors can be conventional and familiar, or conversely, novel and unusual. These linguistic distinctions are substantial because the comprehension of the different types of metaphor entails partially different cognitive strategies and neural substrates. This review gathers studies that have directly investigated which neurocognitive deficits explain the inefficient comprehension of metaphor in schizophrenia. Several impairments have been put forward, such as general intelligence, executive functions and theory of mind deficits. Moreover, the neural correlates of metaphor comprehension in schizophrenia, like the left inferior/medial frontal gyrus and the temporal lobe, match those cortices affected by the neuropathology of schizophrenia. Even though the causal defective mechanism is still a matter of investigation, we provide an attempt to integrate existing findings.

## Introduction

Language disturbances are key signs of schizophrenia syndrome. They are typically grouped under the label “Thought Disorders” (TD). This clinical label was originally introduced to emphasize that schizophrenics’ disorganized speech represents the behavioral manifestation of an underlying anomalous organization of thought, namely the reduced ability to maintain coherence across concepts during speech (Bleuler, 1911/1950). Nonetheless, TD has been used over time to indicate a broader range of schizophrenia linguistic deficits irrespective of their causes (Kuperberg, 2010a). Beyond clinical criteria for language disruption (e.g., derailment, clanging, or poverty of content; Andreasen, 1986), more fine-grained psycholinguistic assessments suggested that several high-order language processes are affected in schizophrenia (Kuperberg, 2010b), such as syntax complexity in production (DeLisi, 2001; Kircher et al., 2005) and comprehension (Perlini et al., 2012), pragmatics (Covington et al., 2005; Perlini et al., 2012), semantics (for a review, Goldberg and Weinberger, 2000), and executive functions supporting language abilities (Bagner et al., 2003).

A sign of schizophrenics’ high-order language comprehension impairment is concretism. Concretism consists in the inability to understand figurative expressions, such as proverbs (for reviews, Mitchell and Crow, 2005; Thoma and Daum, 2006), idioms (for a review, Sela et al., 2015), ironic remarks (Rapp et al., 2013). Figurative language comprehension does not rely on the compositional analysis of lexical items, like literal utterances; if the listener tries to keep the literal meaning, overlooking alternative non-literal ones, the sentence would turn out nonsensical within the given context. While healthy people can effortlessly grasp figurative meanings, schizophrenics may not get rid of the literal meaning, so much that, when asked to paraphrase figurative expressions, they answer providing idiosyncratic and bizarre interpretations (e.g., *locked cry* paraphrased as “a forgotten sadness, like forgetting to pick up child from school, which is very sad,” example from Zeev-Wolf et al., 2015).

In the last decades, studies adopting the neuropsychological approach to concretism have put forward the hypothesis that the deficit is likely to be the secondary effect of more general psychological impairments, such as theory of mind (TOM) anomalies (Gavilán and García-Albea, 2011, 2013), deficits in executive control over language (Titone et al., 2002; Schettino et al., 2010), impaired context processing (Strandburg et al., 1997) and aberrant semantic memory organization (Spitzer, 1997).

Nonetheless, figurative expressions, such as idioms, proverbs, metaphors, differ in terms of semantic and syntactic structure. Therefore, the inefficient comprehension of distinct cases of figurative language may stem from different deficits.

The focus of the present review is to examine metaphor comprehension in schizophrenia. We aim to provide an overview of the existing neuropsychological evidence concerning this aspect of language and its impairment (for a more broader review, see Thoma and Daum, 2006). First, a brief introduction on figurative language in general and metaphor in particular will be provided. Second, the literature concerning the dysfunctional neurocognitive mechanisms underlying metaphor comprehension impairment in schizophrenia will be reviewed. Third, we aim at integrating the available findings. Finally, we suggest future developments on this topic.

## Figurative Language and Metaphor Comprehension

Keeping up with a conversation requires, among several language skills, the ability to comprehend the additional meanings not directly expressed in an utterance, i.e., conversational implicatures (Grice, 1975). The understanding of figurative expressions represents a special case of conversational implicature comprehension; indeed, to infer the communicative intentions of the speaker (i.e., the figurative meaning), the listener is required to go beyond the literal level of the sentence.

Metaphors constitute a special class of figurative expressions whose meaning arises from the semantic overlap between two distant concepts. The first definition comes from Aristotle, who in *Poetics* (1457b) defined metaphor as a substitution of a term with another because of matching attributes. Alternatively, Quintilian in *Institutio Oratoria* (VIII, 6, 8), posited that metaphor is a *similitudo brevior* (abbreviated simile). Basic constituents of a metaphor are the *tenor* and the *vehicle* (Richards, 1936); the *tenor* is the subject of the metaphor, while the *vehicle* is the semantic domain from which certain attributes are selected and assigned to the *tenor*. In cognitive linguistics, other terms have been used, such as *target* and *source* (Kövecses, 2010). According to the standard approach, the interpretation of the metaphor *Dew is a veil*, relies on grasping common attributes between the target *dew* and the source *veil*, such as being transparent, covering and shimmering (Bowdle and Gentner, 2005).

Metaphors can be classified in various way. First, metaphors differ in their level of conventionality, that is, how much their usage is well-established and recurrent in a culture; therefore, metaphors can be either highly conventional (e.g., *Marriage is a jail*) or novel (e.g., *She is a loudspeaker*). A slightly different criterion of categorization is the degree of salience: salient metaphor meaning is very familiar to the individual and already stored in his/her mental lexicon, whereas unfamiliar metaphor meaning is unknown or less prominent to him (Giora, 2002; Bowdle and Gentner, 2005). Second, metaphors can diverge in the grammatical form of the vehicle; accordingly, nominal metaphors express the metaphoric meaning by a noun (e.g., *John is a lion*), predicate metaphors by a verb (e.g., *Paul often runs with his mind*) and adjective metaphors by an adjective (e.g., *Lucy has an open heart*) (Chen et al., 2008; Cacciari et al., 2011; Sakamoto and Utsumi, 2014). Third, metaphors differ in their level of aptness, that is the degree to which the vehicle captures salient attributes of the tenor. Apt metaphors sound as well-constructed expressions because of the clear analogy between the two semantic domains (e.g., *Memory is a warehouse*); conversely, non-apt metaphors may appear inappropriate or less intuitive (e.g., *His spectacles were sandpaper*) (Giora, 2002; Thibodeau and Durgin, 2011).

Cognitive processes underlying metaphor and, more broadly, figurative language, comprehension have been extensively debated. Initially, major attention was devoted to the issue of clarifying whether literal and figurative meanings are processed sequentially (*Standard Pragmatic Model*; Grice, 1975; Searle, 1979) and/or directly (Glucksberg, 2003). However, novel approaches suggest that other parameters, beyond the traditional distinction between literality and figurativeness, are more relevant for metaphor comprehension, i.e., salience and conventionality (Giora, 2002; Bowdle and Gentner, 2005; Jung-Beeman, 2005). The *Graded Salience Hypothesis* (Giora, 2002) suggests that the initial lexical access is ruled by the salience of an expression, regardless of literality/figurativeness. As a general rule, a salient metaphoric meaning would be retrieved more easily than an unfamiliar metaphoric one because it is more prominent into the mental lexicon. However, if the salient meaning is contextually incompatible, it will be suppressed to overcome interpretation inappropriacy.

A further hypothesis, the *Career of Metaphor model* (Bowdle and Gentner, 2005), postulates that conventional and novel metaphors require qualitatively different cognitive processes. Non-conventional metaphors call for an online process of comparison that examines the highest as possible number of attributes of both the vehicle and the tenor in order to reveal structural similarities among concepts (*horizontal alignment*). On the other hand, conventional metaphors require to relate a certain target attribute to a stored abstract schema of meaning directly elicited by the vehicle (*vertical alignment* or *categorization*). As a result, a conventional metaphoric meaning can be accessed faster because it no longer requires the online comparison of semantic domains, but rather the quick retrieval of an overlearned structure of meaning from memory.

The *Fine-Coarse Semantic Coding Hypothesis* (Jung-Beeman, 2005) postulates that the right hemisphere (RH) and the left hemisphere (LH) are specialized in two different processes of semantic spreading activation and integration: coarse semantic coding and fine semantic coding, respectively.

When a word is listened, the LH rapidly triggers a focal semantic field including closely related meanings; meanwhile, the RH activates a widespread semantic field comprising both close and distant meanings. According to this model, conventional metaphors interpretation mainly stems from the LH fine coding, since it allows the quick retrieval of the conventional metaphoric association between the target and the vehicle. On the other hand, the RH coarse coding fundamentally contributes to novel metaphor comprehension by integrating the unusually associated (and hence, distant) terms of the unfamiliar metaphoric expression.

In the last decades, the neural substrates of figurative language comprehension have been broadly explored both in healthy people and clinical populations (for reviews, Thoma and Daum, 2006; Cacciari and Papagno, 2012). Research to date has been concentrated on whether the brain deals with literal and figurative items by recruiting distinct or overlapping neural resources. Even though earlier studies supported a RH unique role for figurativeness processing (e.g., Winner and Gardner, 1977; Bottini et al., 1994), subsequent studies have challenged this view in favor of a more bilateral, predominantly left, involvement (e.g., Oliveri et al., 2004; Papagno et al., 2006; Zempleni et al., 2007; Romero Lauro et al., 2008). Meta-analytic accounts of functional neuroanatomy of figurative language comprehension in healthy people has further confirmed that the comprehension of figurative stimuli at large elicits a bi-hemispheric fronto-temporal network, wherein stronger activation foci are left-lateralized (Bohrn et al., 2012; Rapp et al., 2012). These results are in line with the hypothesis that the LH is involved in figurative meaning processing and that other factors, such as verbal stimuli novelty and complexity, may better explain the extent of the RH recruitment (Coulson and Davenport, 2012). As for metaphors, it has been found that conventional metaphors elicit a LH fronto-temporal activation, while novel metaphors elicit a more bilaterally distributed fronto-temporal network, albeit with stronger left-lateralized activations (Bohrn et al., 2012; Rapp et al., 2012). The extent of the RH involvement could be determined, therefore, by the degree of metaphor unfamiliarity and the RH might be highly involved in capturing overlapping attributes between distant semantic domains during novel metaphor processing (Mashal et al., 2007; Pobric et al., 2008; Faust and Kenett, 2014). Alternatively, the RH recruitment might be driven by stimuli difficulty, which is more dependent on specific task-type demands than on figurativeness or novelty (Yang et al., 2009).

## Metaphor Misinterpretation in Schizophrenia

Here, we review a set of studies which have addressed metaphor comprehension in schizophrenia. In the first part, we summarize all the available literature reporting the experimental approach and major findings of each study. Studies are grouped according to the type of test and neuroimaging techniques used to address the deficit, in the following order: studies that only made used of semantic comprehension tasks, studies that add the assessment of other neuropsychological deficits, and then investigations based on functional magnetic resonance imaging (fMRI), magnetoencephalography (MEG), and electroencephalography (EEG) findings. Finally, we try to integrate reviewed studies.

### Methods

Studies were searched on *PubMED* database by keywords: “metaphor,” “comprehension,” “schizophrenia.” This search provided a total of 17 studies. To be included, studies had to provide an experimental account of the comprehension of metaphors in schizophrenia via behavioral methods and/or via neuroimaging techniques (EEG, fMRI, PET, and MEG). There were no limitations regarding the year of publication. After a first screening, 9 out of 17 studies were excluded because they did not specifically test metaphor comprehension. Then, we examined references of the selected studies and, in this way, we were able to include 13 additional studies; these studies were retrieved from *Scopus* or *Web of Science* databases. A total of 21 studies was finally reviewed (see **Table 1**).

**Table 1:** Main characteristics of the reviewed studies.

	Comparison groups	Metaphor comprehension tasks	Additional neuropsychological tasks	Key findings
Chapman, 1960	51 SZ 41 Brain damaged 36 Medical/surgical neurotypical patients	• *Multiple-choice paraphrasing task*: choose the correct interpretation of literal and metaphoric sentences among three alternatives (literal, metaphoric, and unrelated interpretation sentences).	–	SZ tend to interpret metaphor literally (“*antifigurative bias*”).
Cutting and Murphy, 1990	20 SZ^a^ 20 Manic 20 Depressed	• *Triads test*: choose the two words that are most similar in meaning or that go together best from a pool of three presented words.	–	SZ tend to select word pairs according to their denotative meaning (“*denotative bias*”).
Spitzer et al., 1994	35 SZ^?^ 43 HC	• *Lexical decision task*: decide whether a target word, presented after a metaphoric statement, is a word or a non-word. Target word was concretely, metaphorically, or non-related.	–	SZ demonstrate larger concrete priming and absence of abstract priming effect.
Iakimova et al., 2006	25 mixed SZ^a^ 18 Depressed 22 HC	• *Multiple-choice paraphrasing task*: choose the correct interpretation of metaphoric sentences among four words related to the figurative, the literal, the concrete, and an unrelated meaning.	–	SZ tend to select the concrete and the literal interpretation option. *Context processing is influenced by the concreteness bias and the literality bias*.
Elvevåg et al., 2011	21 SZ^a^ 21 HC	• *Free paraphrasing task*: provide the meaning of the presented emotionally-laden metaphoric sentence.	–	SZ, as well as HC, are less prone to literally interpret emotionally-laden metaphors. *Emotion does not increase schizophrenics’ literality bias*.
Chakrabarty et al., 2014	16 SZ^?^ 16 HC	• *Multiple-choice semantic judgment task*: judge if the presented word pairs (Lit, NM, CM, and UR) are literally related, metaphorically related, or unrelated. • *Multiple-choice sentence completion task*: complete presented sentences (Lit, NM, CM, and Amb) by choosing the appropriate word from a list of four target words.	–	SZ accuracy in NM and CM comprehension is lower; SZ comprehension increases in sentence completion, mostly with CM. *Novelty, but not context processing, is impaired in schizophrenics’ metaphor comprehension*.
Zeev-Wolf et al., 2014	17 Thought-disordered SZ^?^ 30 HC	• *Semantic judgment task in split visual field presentation*: decide whether the presented word pairs (Lit, NM, CM, and UR) are meaningful.	–	SZ accuracy in CM and UR pair comprehension is lower, but higher in NM pair. In HC, CM: LH advantage at both SOAs; NM: LH advantage at short SOA and RH advantage at long SOA; UR: LH advantage at both SOAs. In SZ, CM: RH advantage at both SOAs; NM: RH advantage at short SOA and LH advantage at long SOA; UR: RH advantage at both SOAs. *Overabundance of RH coarse semantic coding; salience processing is impaired in schizophrenics’ metaphor comprehension.*
Drury et al., 1998	1° Comparison: 14 Mixed SZ^a,b^ 10 Psychotic non-SZ^a,b^ 12 Depressed^a,b^ 2° Comparison: 21 Deluded^a,b^ 12 Non-deluded^a,b^	• *Multiple-choice sentence completion task*: complete presented sentences (simile and metaphor) missing the last word by choosing from a list of six words. • *Metaphor/Irony embedded in a story task*: answer to open-ended questions about the meaning of ironic and metaphoric sentences uttered by a character.	• ToM: second-order false belief task.	SZ equally perform on metaphoric sentence completion task, but they are less able to comprehend metaphoric sentence embedded in a story. Deluded and non-deluded patients similarly perform on both metaphor comprehension tasks.
Herold et al., 2002	20 Paranoid^b^ 20 Non-psychiatric	• *Metaphor/Irony embedded in a story task*: answer to open-ended questions about the meaning of ironic and metaphoric sentences uttered by a character.	• ToM: (a) First-order false-belief task; (b) Second-order false-belief task.	Paranoids equally perform on metaphoric sentence completion task.
Langdon et al., 2002	25 SZ^a+b^ 20 HC	• *Metaphor/Irony embedded in a story task*: judge whether the meaning of ironic and metaphoric sentences uttered by a character is appropriate in that situation.	• ToM: picture sequencing\false-belief stories Executive: (a) Picture sequencing\capture stories; (b) Tower of London task. • General Intelligence: (a) Picture sequencing\mechanical stories; (b) Spot-the-Word test; (c) Digit-forward/backward WAIS subtest.	SZ are less accurate in comprehend metaphor embedded in a story. Score at executive tests predicts performance on ToM and metaphor comprehension. Partialling out all the measures of general intelligence and executive functioning in the regression analysis, ToM performance does not predict metaphor comprehension. *ToM deficit does not contribute to schizophrenics’ metaphor misinterpretation. Other deficits (beyond the executive dysfunction) underlie the misinterpretation*.
Champagne-Lavau and Stip, 2010	20 SZ^a^ 20 HC	• *Free metaphoric sentence paraphrasing task*: provide the meaning of the presented metaphoric sentence (NM, CM); if unable, choose the correct interpretation via multiple-choice (the literal, the metaphoric, and an incongruent interpretation).	• ToM: first-order and second-order false-belief task. • Executive: (a) Stroop test; (b) Hayling test; (c) Trial Making Test; (d) Wisconsin Card Sorting Test.	SZ are less able to interpret NM and CM. Using ToM score as covariate, SZ were still less competent at NM; using Trial Making-B and Wisconsin Card Sorting test scores as covariates, SZ were still less competent at NM and CM. *ToM deficit contributes to schizophrenics’ misinterpretation of CM*.
Mossaheb et al., 2014	40 Mixed SZ^a+b^ 43 HC	• *Novel metaphor triads test*: make as many pairings as possible from a pool of three presented words/sentences and explain the decision. • *Free paraphrasing/Multiple-choice metaphoric proverb comprehension task*: freely explain the meaning of the half of the presented items; choose the interpretation from a list of four option for the other half of the items.	• Executive: (a) Stroop test; (b) Trial Making Test; (c) Wisconsin Card Sorting Test. • Verbal Intelligence: (a) information; (b) comprehension; (c) digit span; (d) vocabulary; (e) arithmetic; (f) similarities. (i.e., WAIS subtests). • Non-verbal Intelligence: Raven’s Standard Progressive Matrices.	SZ are less able to comprehend NM and metaphoric proverbs. According to multiple regression analysis, Trial Making-B largely predict metaphoric proverb comprehension, whereas vocabulary WAIS subtest largely predict the ability to generate NM. *Cognitive flexibility contributes to schizophrenics’ misinterpretation of CM, whereas the level of vocabulary to NM*.
Mo et al., 2008	29 Mixed SZ^b^ 22 HC	• *Metaphor/Irony embedded in a story task*: answer to open-ended questions about the meaning of ironic and metaphoric sentences uttered by a character.	• ToM: (a) First-order false-belief task; (b) Second-order false-belief task • General Intelligence: WAIS (general IQ) • Verbal Intelligence: WAIS (verbal IQ)	SZ result to be less able to comprehend metaphoric sentence embedded in a story in ANCOVA with general IQ as covariate. By comparing SZ and HC subgroups matching for verbal IQ, SZ are still less able. According to multiple regression analysis, second-order ToM and Group predictors account for variance of metaphor comprehension. *ToM deficit and IQ decline do not strongly contribute to schizophrenics’ metaphor misinterpretation*.
Varga et al., 2014	19 Paranoid^b^ 19 HC	• *Metaphor embedded in a story task*: answer to open-ended questions about the meaning of NM and CM sentences uttered by a character.	• General intelligence: WAIS	Higher-IQ Paranoids are as able as HC to comprehend NM and CM sentences embedded in a story. Lower-IQ Paranoids are less able to comprehend NM only. *Poor IQ contribute to schizophrenics’ metaphor misinterpretation*.
Tavano et al., 2008	37 SZ^b^ 37 HC	• *Free metaphoric sentence paraphrasing task*: provide the meaning of the presented CM and opaque idiomatic sentences.	• Syntax: [Test di Comprensione Grammaticale].	SZ are less able to comprehend CM and idioms. Comprehension of metaphor correlates with syntactic correlation. *Reduced access to structural syntactic frames contribute to schizophrenics’ metaphor misinterpretation*.

	**Comparison groups**	**Metaphor comprehension tasks**	**Technique**	**Key findings**

Iakimova et al., 2005	20 mixed SZ^a+b^ 20 HC	• *Semantic judgment task in rapid serial visual presentation*: decide whether the presented sentences (literal, metaphoric, incongruous) are meaningful.	Electroencephalography	SZ are less able to judge both literal and metaphorical sentences. SZ show higher N400 in all sentence type. C*ontext processing is impaired in schizophrenia*.
Kircher et al., 2007	12 SZ^b^ 12 HC	• *Valence judgment task*: judge whether the presented sentences (literal and metaphoric) have a positive or a negative connotation.	Functional magnetic resonance imaging	SZ abnormally engage the left IFG and poorly recruit the right MTG and STG during metaphor comprehension.
Mashal et al., 2013	14 SZ^b^ 14 HC	• *Semantic judgment task*: judge whether the presented word pairs (MN, MC, Lit, and UR) are meaningful.	Functional magnetic resonance imaging	SZ strongly engage left MFG (instead of right IFG and SFG) during NM and CM comprehension. SZ even more strongly activate left MFG during NM comprehension.
Mashal et al., 2014	12 SZ^b^ 12 HC	. • *Semantic judgment task*: judge whether the presented word pairs (MN, MC, Lit, and UR) are meaningful.	Functional magnetic resonance imaging	SZ show an abnormal functional connectivity between the precuneus and fronto-temporal cortices.
Zeev-Wolf et al., 2015	10 SZ^?^ 14 HC	• *Semantic judgment task in split visual field presentation*: decide whether the presented word pairs (Lit, NM, CM, and UR) are meaningful.	Magnetoencephalography	SZ show an abnormal RH fronto-temporal activation in response to linguistic stimuli (Lit, NM, CM, and UR) at 170 ms latency. *Overabundance of RH coarse semantic coding since earlier stages of semantic processing*.

*Mixed SZ = mixed group of different schizophrenia subtype (disorganized, paranoid, undifferentiated) and/or schizoaffective disordered patients; a = inpatients or in acute phase; b = outpatients or in remission; ? = not specified; Lit = literal; NM = novel metaphoric; CM = conventional metaphoric; Amb = ambiguous; UR = unrelated.*

### Behavioral Accounts of Metaphor Misinterpretation

A fairly large body of literature concerns cognitive impairment underlying the difficulty that people with schizophrenia encounter when facing a metaphor. Present evidence stems from different behavioral paradigms and theoretical frameworks developed over the years. For the sake of completeness, we report here the available findings.

The first study on metaphor misinterpretation in schizophrenia dates back to Chapman (1960), who addressed the proneness of schizophrenic individuals toward literal vs. figurative interpretation of metaphor. Chapman asked participants to read literal (e.g., *Miss Bailey’s illness turned her yellow*) or metaphoric (e.g., *David turned yellow when he faced the enemy*) statements and to choose the correct interpretation among a literal (i.e., *His/Her skin became discolored*), a metaphoric (i.e., *He/She become cowardly*), and an unrelated (i.e., *He/She become hungry*) option. Evaluation on error trend with respect to controls demonstrated that schizophrenics more frequently paraphrased metaphors literally and less frequently interpreted literal sentences metaphorically, showing an opposite tendency with respect to brain-damaged patients with mixed neurological diagnoses. Later, Cutting and Murphy (1990) based their investigation on the denotative-connotative distinction. Denotation refers to the most typical meaning, while connotation refers to a subordinate meaning. In their metaphor comprehension task, participants were presented with triads of words they had to group in pairs according to either the denotative meaning (i.e., semantic domain, e.g., *foolish* and *loving*; antonym, e.g., *cold* and *warm*) or the connotative meaning (i.e., metaphor, e.g., *cold* and *hateful*; polarity, e.g., *shallow* and *hateful*) of words. Schizophrenics showed a stronger tendency to select pairs based on both types of denotative meaning (semantic domain and antonym) suggesting that the denotative bias formulation better captures the very essence of the comprehension deficit.

Overall, these first studies experimentally proved concretism for metaphors, providing further accounts of the figurative language deficit beyond the more broadly studied misinterpretation of proverbs (Thoma and Daum, 2006). Moreover, they provide the first phenomenological descriptions of schizophrenics’ metaphor comprehension impairment.

More recently, however, researchers became interested not only on the type of impairment found in schizophrenics but also on the underlying mechanism. In the first pool of studies reviewed below, only metaphor comprehension tasks were used. Various facets of semantic processing are investigated, such as semantic spreading into mental lexicon (Spitzer et al., 1994), novelty of metaphor (Zeev-Wolf et al., 2014), semantic integrative processes required to handle contextual information (Iakimova et al., 2006; Chakrabarty et al., 2014), and finally emotional connotation (Elvevåg et al., 2011).

The first hypothesis put forward suggested that schizophrenics’ preference for concrete meaning of words stems from abnormal processes and/or aberrant structure in the semantic memory (Anand et al., 1994; Spitzer, 1997; Goldberg and Weinberger, 2000), together with limitations in working memory (Spitzer et al., 1994). Spitzer et al. (1994) assessed semantic spreading into mental lexicon with a semantic priming decision task. During this task, participants listened to a set of metaphoric sentences that were used to prime target words that could correspond either to the metaphoric, concrete (i.e., related to a single word in the sentence) or an unrelated interpretation. While healthy subjects showed either metaphoric or concrete priming effects, schizophrenics displayed only concrete priming. Spitzer et al. (1994) interpreted their results according to earlier models of language comprehension. These models suggest that figurative meaning processing takes place only after the literal meaning has been rejected because it has been judged implausible in a given context (Grice, 1975; Searle, 1979). Because of the sequential nature of this process, metaphor interpretation requires additional cognitive resources in comparison to literal meaning. Based on this theoretical framework, Spitzer et al. (1994) suggested that working memory deficits could prevent schizophrenic patients to retain online lexical items until later stages of meaning processing, leading schizophrenic people to accept the literal meaning.

The role of salience in metaphor was instead the focus of interest in Zeev-Wolf et al.’s (2014) study. The authors predicted that, because of the failure of LH lateralization for language areas (Crow, 1997), schizophrenics’ semantic coding would be predominantly coarse (i.e., RH-dependent). As a result, novel metaphor comprehension would be facilitated in schizophrenic patients. In this study, the visual hemifield technique was combined to the semantic priming procedure: after the priming word (e.g., *juicy*) was displayed in the center of the screen, the target word (e.g., *gossip*) appeared to the right or the left visual field, with 250 or 750 ms stimulus onset asynchrony (SOA). Participants had to judge whether the word pair was meaningful. Presenting the target word in one of the visual hemifield provides the contralateral hemisphere with a relative advantage in processing the word pair. The comparison between performances relative to each hemisphere allowed quantifying the extent of coarse vs. fine semantic coding with respect to different types of metaphor. The two SOAs permitted probing the prominence of coarse vs. fine semantic coding at earlier and later phases of processing. As predicted, TD schizophrenic patients underperformed healthy participants with respect to all stimulus types (literal, conventional metaphoric, and unrelated pairs), but outperformed relative to novel metaphors. This result was coupled with a consistent over-reliance on RH coarse coding across conditions. The authors argued that this phenomenon entails two effects. On the one hand, subordinate literal meanings are uselessly active during conventional metaphors comprehension. On the other hand, distant unfamiliar associations are readily activated during novel stimuli comprehension. This latter, however, represents an advantage with novel meaningful (i.e., novel metaphors), but a disadvantage with novel nonsensical (i.e., unrelated pairs) stimuli. The abnormal coarse coding implies a broken interplay between RH and LH that makes LH unable to suppress irrelevant or meaningless associations (Pobric et al., 2008). As a result, the ability to discriminate between novel meaningful (i.e., novel metaphors) and novel nonsensical (i.e., unrelated pairs) expressions is significantly limited.

Another study (Chakrabarty et al., 2014) examined the effect of salience on metaphor comprehension in schizophrenia also considering the effect of sentence context. To address these two issues, novel and conventional metaphor comprehension was evaluated both in minimal context and in sentence context. First, participants performed a semantic judgment task on two-words stimuli (e.g., novel metaphor pair: *sweet pain*), where they had to judge if the two words were metaphorically related, literally related or unrelated. Second, they accomplished a sentence completion task by selecting proper targets of literally biased, metaphorically biased or unbiased sentences (e.g., novel metaphor biased: *The sweet [pain] of motherhood is every woman’s dream*). Results demonstrated that schizophrenics were, overall, less able to deal with both conventional and novel metaphors. However, schizophrenics’ performance improved on the sentence completion task, with a larger increase for conventional metaphors. These results stress that sentence context processing is intact in schizophrenia because it improves metaphor comprehension, with a stronger effect of facilitation on the retrieval of conventional metaphoric meanings. By using a different methodological approach, Iakimova et al. (2006) investigated which type of context processing mostly occurs in schizophrenia when these patients are presented with metaphoric sentences. The authors argued that sentence processing might be affected by two different tendencies in schizophrenia, namely the concreteness bias (i.e., anchoring to a single word in the sentence) and the literality bias (i.e., whole-sentence processing biased toward word-by-word analysis). In their study, participants were invited to read familiar metaphoric sentences (e.g., *That sound makes my blood run cold*) and to select the correct answer among a list of four words related to the figurative (e.g., *fright*), the concrete (e.g., *red*), the literal (e.g., *freezing*), or an unrelated (e.g., *flower*) interpretation of the sentence. Schizophrenics, as depressed patients, showed a higher percentage of both literal and concrete misinterpretations than controls, indicating that literality bias and concrete bias co-occur in these psychiatric populations.

Finally, Elvevåg et al. (2011) hypothesized that the retrieval of the metaphoric meaning might be affected by the difficulty to deal with emotionally-laden concepts in schizophrenia. Participants, therefore, were asked to paraphrase metaphors that could have emotional (e.g., *He was burning with desire*) or neutral (e.g., *His brain is a computer*) connotation. The emotional connotation more frequently induced the metaphoric interpretation in both schizophrenics and healthy controls; however, schizophrenics still produced more literal interpretations. In our view, these results suggest that an emotional content in metaphors does not increase or decrease schizophrenics’ tendency toward literality.

Differently from the studies reported above, the following ones searched for direct and overt evidence of the association between cognitive deficits and impaired metaphor processing. To this aim, additional neuropsychological tests were administered besides a metaphor comprehension task. In general, these studies investigate whether deficits of ToM, intelligence quotient (IQ) and executive functions have an impact on the comprehension of metaphor in schizophrenia.

Theory of mind is the ability to infer the content of others’ mental states, i.e., their beliefs and intentions. ToM tasks usually explore two different inferential abilities: the acknowledgment of a character’s belief about the world in a short story (i.e., first-order false beliefs) and the acknowledgment of what a character thinks about another character’s thoughts (i.e., second-order false beliefs).

In the wake of the finding that ToM abilities may strongly predict the comprehension of metaphor and irony in autism (Happé, 1993), ToM assessment has been carried out together with the evaluation of pragmatic communication deficits also in schizophrenia. Happé’s findings, specifically, suggested a links between metaphor comprehension and first-order ToM (i.e., the ability to infer the speaker’s intention to convey a non-literal meaning) and between irony comprehension and second-order ToM (i.e., the ability to recognize the speaker’s intention to express the opposite of the apparent meaning of the utterance).

A couple of studies used irony and metaphor comprehension performances as indirect evidence of ToM impairments in schizophrenia (Drury et al., 1998; Herold et al., 2002). Drury et al. (1998) tested the hypothesis that during an acute episode of schizophrenic symptoms, patients would show both first-order and second-order ToM deficits, whereas patients suffering from paranoid symptomatology would specifically present second-order ToM problems. First, patients were asked to comprehend ironic and metaphoric statements embedded in a story context. Second, they performed a metaphor sentence completion task, in which they had to choose the item that completed the presented metaphoric sentence from a list of words (e.g., *The dancer was so graceful. She really was… [a swan]*). Schizophrenics in acute phase performed as good as the control groups on the metaphor sentence completion task. As for the second task, they poorly understood metaphors, but they did not show difficulties with irony comprehension. In the authors’ view, this scarce comprehension of metaphors cannot be the effect of a ToM dysfunction. Indeed, if first-order ToM competence underlies metaphor comprehension, the poor understanding of metaphor would not be consistent with the spared higher-order ToM, proved by good comprehension of irony. As for paranoid symptoms, they were not associated to an impairment either in metaphor or irony comprehension.

Herold et al. (2002), instead, investigated whether ToM impairment recovers with the remission of acute paranoid symptoms. Remitted patients were found to properly interpret metaphors embedded in a story, but to poorly understand irony. In Herold et al.’s (2002) view, these findings are indicative of preserved first-order ToM, but defective second-order ToM in paranoids in remission. It should be noted, however, that the two foregoing studies used control groups composed by patients suffering from other psychiatric diseases and by vaguely defined non-psychiatric subjects, respectively. The absence of healthy controls might have prevented the schizophrenics’ metaphor comprehension deficit to clearly emerge.

Other factors have been considered to explain metaphor misinterpretation in schizophrenia, namely reduced executive control and general intelligence decline. In Langdon et al. (2002), schizophrenic patients were found to be less able than healthy participants to judge the appropriateness of metaphoric and ironic utterances embedded in a story context. Importantly, false-belief picture sequencing score significantly predicted irony but not metaphor comprehension accuracy when inhibitory suppression (measured with capture picture sequencing task), executive planning (Tower of London test) and general intellectual capacity (digit forward-backward; Spot-the-Word test) were taken into account. Hence, ToM impairments did not crucially contribute to the poor comprehension of metaphor in the schizophrenic group. The authors argued that a different cognitive dysfunction, beyond the executive deficit, is more likely to underlie metaphor misinterpretation, i.e., a semantic impairment (Goldberg and Weinberger, 2000).

Focusing on ToM and executive deficits, Champagne-Lavau and Stip (2010) asked participants to orally provide the meaning of novel and conventional metaphoric sentences (e.g., *This bus is a turtle* – *My friend has a heavy heart*). Schizophrenic patients underperformed healthy controls in comprehension of both novel and conventional metaphors. These between-group differences remained significant when controlled for time at the Trial Making Test B, number of perseveration errors and number of completed categories at the Wisconsin Card Sorting Test. However, differences disappeared when adjusted by ToM (ascertained with first- and second-order false-belief questions). In this study, therefore, ToM disabilities were considered to contribute to difficulties with conventional metaphor in schizophrenia. Conversely, the lack of set shifting skills and the poor cognitive flexibility were excluded from major factors.

By using different metaphor tasks, Mossaheb et al. (2014) investigated the relationship between several measures of frontally mediated functions, analogical thinking and verbal intelligence and the comprehension of metaphor in schizophrenia. To evaluate novel metaphor comprehension, they used triads of verbal items to associate in pairs. Participants had to recognize as many meaningful pairs as possible and then explain the meaning of the found associations. Possible combinations could depend on an affective association (e.g., *woman crying – willow tree*), perceptual analogy between concepts (e.g., *sunflower – tall thin woman*) and category-overlapping similarities (e.g., *snorting bull – man boxing*). Performance on this task was lower in the schizophrenic group and largely predicted by Wechsler’s vocabulary subtest. A second task focused instead on the comprehension of conventional metaphoric sentences (i.e., proverbs; e.g., *We are all in the same boat*) to interpret via free paraphrasing or via multiple-choice. Performance on this task was also poorer in schizophrenic subjects, however, it was largely predicted by time at Trial Making Test B, used in the study to ascertain cognitive flexibility. Moreover, patients’ performance on proverbs multiple-choice task was significantly correlated with Wechsler’s verbal IQ subtests, Raven’s matrices test, Trial Making Test and Wisconsin Card Sorting Test. Overall, these results suggest that difficulties to comprehend metaphors are signs of a more generalized cognitive impairment. At a speculative level, the authors argued that these difficulties might be part of a broader executive dysfunction stemming from the well-known neuropathological abnormalities in frontal cortices in schizophrenia.

General intelligence was controlled for in a couple of studies. This procedure should allow evaluating to which extent schizophrenics’ cognitive disorders affect metaphor comprehension. In Mo et al.’s (2008) study, remitted schizophrenic patients understood metaphoric utterances embedded in a story context less successfully relative to healthy controls, even when the comparison was carried out on subgroups of matching verbal IQ. In addition, accuracy in metaphor interpretation was significantly predicted by second-order false-belief questions; conversely, neither first-order false-belief questions nor general IQ were significant predictors. Even though such results statistically accounted for an association between ToM deficits and metaphor performance, the authors excluded that poor mind-reading is strongly involved in metaphor interpretation deficit in schizophrenia. Conversely, Mo et al. (2008) suggested that, because metaphors and ToM tasks are highly demanding in terms of semantic processing, a higher-order deficit in semantics is likely to account for the observed poor performances on both tasks.

Similarly, Varga et al. (2014) assessed metaphor comprehension and IQ. They recruited only paranoid subtype patients with a normal IQ to increase the homogeneity of the experimental group. Salience of metaphor was also considered by assessing comprehension of conventional and novel metaphor embedded in a story. To take into account the IQ level, the patient group was split into higher-IQ and lower-IQ subgroups. Higher-IQ paranoids demonstrated to be as proficient as healthy controls in novel and conventional metaphor comprehension. Conversely, lower-IQ paranoids showed only a preserved conventional metaphor comprehension. In Varga et al. (2014) view, the preserved general cognitive abilities would permit novel metaphors understanding in higher-IQ patients because they compensate for the ToM deficit. Conversely, unimpaired semantic processing (as indicated by the observation of good comprehension of literal items) might explain the good accuracy demonstrated in conventional metaphor comprehension in both lower-IQ and higher-IQ patient subgroups.

Finally, we mention the study of Tavano et al. (2008) who examined the relationship between metaphor and syntactic comprehension in schizophrenia. With respect to healthy controls, patients provided significantly less appropriate paraphrases of conventional metaphors and opaque idioms (i.e., highly conventionalized figurative expressions, with only a remote relationship between literal and figurative meaning; Cacciari and Papagno, 2012). However, in this study a verbal explanation was required and production definitely involves executive control. Since the level of metaphor and idiom comprehension correlates with the accuracy in syntactic comprehension, the authors suggested that that poorer syntactic comprehension may partially underlie misinterpretation of idiom and metaphor. This is similar to what has been found by Papagno et al. (2004) in aphasic patients. Indeed, difficulties in idiom comprehension in these patients seemed to be due to the fact that they rely on a literal first strategy, accessing a figurative interpretation only when the linguistic analysis fails to yield acceptable results.

### Neurophysiological and Neurofunctional Accounts of Metaphor Misinterpretation

Up to now, limited work has been carried out by means of neurophysiological and neuroimaging methodologies. In this section we report results based on fMRI, EEG, and MEG data.

An ERP study (Iakimova et al., 2005) dealt with the processing of semantic context in schizophrenia with respect to conventional metaphoric literally implausible, literal, or incongruous endings of sentence (e.g., metaphor: *He is away in the… clouds*) displayed on a screen in rapid serial visual presentation. Participants were required to judge the meaningfulness of items. Iakimova et al. (2005) focused on the N400 component because this is linked to the ability to infer the final word of a sentence from the sentence context. Schizophrenic patients showed lower accuracy, higher reaction times and larger N400 amplitude than healthy controls in all types of sentences. The N400 pattern suggested that schizophrenic patients are less able to integrate pieces of information forming the semantic context of a sentence; however, this problem is not specific for metaphor comprehension, but affects metaphoric as well as literal language comprehension.

Kircher et al. (2007) analyzed the neurofunctional correlates of metaphor comprehension of participants accomplishing a valence judgment task (i.e., to judge the positive or negative connotation of the sentence) with novel metaphors (e.g., *The lovers words are harp sounds*) and literal sentences (e.g., *The lovers words are lies*). The contrast between novel metaphor and baseline conditions compared between groups showed that schizophrenics activate to a major extent the left inferior frontal gyrus (IFG), but to a lesser degree the right middle and superior temporal gyrus (MTG and STG). Moreover, the maximum activation in the left IFG was more dorsally located than in controls and correlated with severity ratings of abstract thinking. Kircher et al. (2007) hypothesized that concretism may arise from an abnormal engagement of the left IFG and a poor recruitment of the right temporal cortex.

A more recent study (Mashal et al., 2013) suggested that schizophrenics might abnormally engage left frontal cortices to deal with novelty of metaphors. To explore the neural substrates of the ability to map semantic associations between words, Mashal et al. (2013) presented literal (e.g., *birth weight*), novel metaphoric (e.g., *hatred net*), conventional metaphoric (e.g., *sharp tongue*) or meaningless (e.g., *road bees*) two-word stimuli. Participants had to silently judge the pairs meaningfulness. The effect of *metaphoricity* of novel metaphors was associated to a different pattern of activation enhancement in the two groups: the right IFG and the right SFG in controls, whereas the left MFG, the left inferior parietal lobule and the left precuneus in schizophrenic individuals. The additional activation of the left MFG and parietal cortices in schizophrenia would represent the need for compensative cognitive strategies to increase the comprehension of figurative aspects of stimuli. A left-lateralized abnormal recruitment was also detected for *novelty*: healthy controls were found to more strongly engage the right IFG and SFG to process metaphor novelty, whereas schizophrenics the left MFG. The authors suggested that this latter finding might correspond to the additional working memory resources that schizophrenics employ to process the more demanding novel metaphors as compared to conventional metaphors. A further study (Mashal et al., 2014) highlighted that in schizophrenics, but not in controls, there was a significant functional connectivity between the precuneus and fronto-temporal cortices, suggesting that precuneus activation in schizophrenia might provide mental imagery support during both literal and novel metaphor comprehension.

Zeev-Wolf et al. (2015) used MEG to obtain a more fine-grained account of the time course of neural activity during metaphor comprehension in schizophrenia. In this study participants carried out a semantic decision task in visual hemifield presentation (as in Zeev-Wolf et al., 2014). As previously found (Zeev-Wolf et al., 2014), schizophrenics outperformed healthy participants in judging the meaningfulness of novel metaphors, but they were less accurate with literal, conventional metaphoric and unrelated pairs. MEG data showed that patients overactivated the right fronto-temporal cortex at earlier stages of semantic processing with all stimulus types, providing evidence for a disrupted early-stage LH dominance and inefficient inter-hemispheric interplay. With respect to healthy controls, novel metaphor comprehension elicited a stronger right-lateralized fronto-temporal activation, whereas unrelated pairs elicited a stronger right fronto-temporal activation together with a left frontal activation. As commented by the authors, RH overactivation might indicate a strong proneness to coarse semantic coding, i.e., an abnormal integration of distantly-related semantic concepts. This proneness to coarse coding is advantageous in the case of meaningful pairs (i.e., novel metaphor), but imposes a higher effort for deciding the meaningfulness (i.e., bilateral frontal areas recruitment) in the case of meaningless pairs (i.e., unrelated pairs).

### Psychopathology and Metaphor Misinterpretation

Several studies report correlations between the symptomatology and the level of metaphor comprehension.

Concretism is clinically assessed by proverbs comprehension questions (Kay et al., 1987). Two fMRI studies found that the abnormal recruitment of left IFG/MFG during metaphoric stimuli processing negatively correlates with the score of concrete thinking severity (Kircher et al., 2007; Mashal et al., 2013). Comprehension of conventional metaphor negatively correlates with concretism in a couple of studies (Mashal et al., 2013; Piovan et al., 2016); no correlations was found for novel metaphors (Mashal et al., 2013). This distinct pattern of correlation is not surprising given that the assessment of concretism consists in the comprehension of conventional linguistic stimuli.

Iakimova et al. (2006) noted that, those patients who showed concreteness and literality bias, obtained a higher general rating of TD (assessed by means of the *Scale for Thought, Language and Communication disorders*; Andreasen, 1979). By separately considering negative TD (e.g., poverty of speech, blocking and increased latency) and positive TD (e.g., derailment, tangentiality, and incoherence), Langdon et al. (2002) found metaphor misinterpretation to be associated only to negative signs of TD.

More interestingly, metaphor comprehension performance turned out to be negatively correlated with negative symptoms (Langdon et al., 2002; Mossaheb et al., 2014) such as apathy and anhedonia (Langdon et al., 2002). However, not all studies detected these significant correlations (Tavano et al., 2008; Champagne-Lavau and Stip, 2010; Zeev-Wolf et al., 2014).

Siddi et al. (2016) directly searched for associations between figurative language impairment and auditory verbal hallucinations in a large group of psychotic patients (both schizophrenia spectrum disorders and bipolar disorders), controlling for the confounding effects of frontal abilities. They found that propensity to suffer from verbal auditory hallucinations could be moderately predicted by their capability to understand metaphor, but not idiom.

### Putative Mechanisms Underlying Metaphor Misinterpretation

Schizophrenia is characterized by several neuropathological changes in both cortical and subcortical areas, such as fronto-temporal cortices, the thalamus, the amygdala and the hippocampus (Maggioni et al., 2016), together with white matter disruptions (Brambilla et al., 2005; Andreone et al., 2007; Corradi-Dell’Acqua et al., 2012). These abnormalities are believed to constitute the neuroanatomical basis of the widespread neuropsychological impairment demonstrated in individuals with schizophrenia (Heinrichs and Zakzanis, 1998; Vöhringer et al., 2013; Bortolato et al., 2015). From this perspective, the general neurocognitive dysfunction may severely affect the capability to successfully undertake metaphoric language processing in schizophrenia. To provide a more detailed outline of the specific cognitive mechanisms underlying metaphor comprehension impairment, in the current section we compare major findings from studies reviewed above. Importantly, several aspects of metaphor comprehension in schizophrenia are still unclear and deserve further investigation; therefore, our conclusions are speculative.

As rather consistently reported, while metaphors can be intuitively understood by healthy people, they pose greater cognitive demand to schizophrenic subjects. Broadly speaking, schizophrenics struggle to understand both novel and conventional metaphors (Champagne-Lavau and Stip, 2010; Mashal et al., 2013, 2014; Chakrabarty et al., 2014; Mossaheb et al., 2014; Varga et al., 2014). Emerging evidence, however, has pointed to different deficits underlying misinterpretation of conventional and novel metaphors (Mashal et al., 2013, 2014; Chakrabarty et al., 2014; Varga et al., 2014; Zeev-Wolf et al., 2014, 2015).

A first account supporting this claim is provided by neurofunctional findings. Schizophrenics demonstrated a stronger activation of the left MFG during novel metaphor compared to conventional metaphor comprehension. This pattern of activation is abnormally left-lateralized with respect to the enhanced recruitment of the right IFG and SFG observed in neurotypical individuals. The increased left MFG activation elicited by novel metaphors in schizophrenic individuals may thus represent the neural substrate of an inefficient strategy adopted to deal with the higher cognitive demand made by novelty as compared to conventionality of metaphors (Mashal et al., 2013). Similarly, Kircher et al. (2007) found that comprehension of non-salient metaphors in schizophrenia is characterized by an abnormal activation of the left frontal cortex, together with a weaker engagement of the right MTG/STG. Nonetheless, these first findings do not allow a direct comparison of novel and conventional metaphors (i.e., all metaphors were non-salient). In addition, differently from Mashal et al. (2013) these results are based on sentence stimuli; therefore, they do not allow ruling out the role of semantic processing elicited by the sentence context.

As demonstrated in Zeev-Wolf et al. (2015), semantic processing in schizophrenia is characterized by a greater magnetoencephalographic component in the RH fronto-temporal regions. RH overactivation seems therefore to constitute the neural substrate for an excessive semantic integration between presented words and related concepts (i.e., over-abundance of coarse semantic coding), which is demonstrated by the apparent competence with novel metaphorical stimuli and by the limited ability to recognize novel meaningless stimuli.

Despite different spatial and temporal resolutions strongly limit the comparison between different techniques, fMRI and MEG findings concerning novel metaphor comprehension can provide complementary evidence. We speculatively suggest that the hyperactivation of the left frontal cortex observed in fMRI studies (Mashal et al., 2013, 2014) might reflect the abnormal need for selection processes (e.g., working memory load and/or inhibitory suppression) in trying to manage the exaggerated amount of semantic associations produced by the dysfunctional early-stage RH overactivation (Zeev-Wolf et al., 2014, 2015).

Support for the idea that the impairment of novel and conventional metaphor comprehension is heterogeneous comes also from neuropsychological studies, because they overall suggest that different cognitive mechanisms underlie the reduced comprehension of these two types of metaphor.

Varga et al. (2014) found that, among individuals of normal general intelligence (measured by means of WAIS), those with lower levels of general intelligence poorly comprehended novel metaphors, but still understood conventional metaphors. These findings, thus, suggest the need for higher cognitive competence to process novel metaphors. A number of studies (Champagne-Lavau and Stip, 2010; Mossaheb et al., 2014) also looked for different cognitive measures accounting for distinct causative impairments. In a study (Mossaheb et al., 2014), the comprehension of metaphoric proverbs was linked to cognitive flexibility skills and the ability to detect novel metaphors was associated to the level of vocabulary (measured by the dedicated Wechsler’s subtest). Conversely, in another study (Champagne-Lavau and Stip, 2010) cognitive flexibility and set shifting (considered executive functions, that the authors assessed by means of the Trial Making Test and Wisconsin Card Sorting Test) were not found to significantly contribute either to schizophrenics’ impaired comprehension of novel or conventional metaphors. Findings concerning conventional metaphors are hard to integrate given the overtly divergent results related to cognitive flexibility. Nonetheless, it could be argued that other executive functions tests, such as working memory tests, could have better controlled for schizophrenics’ metaphor comprehension because low working memory abilities might prevent schizophrenic patients to maintain the verbal string online while retrieving the metaphoric meaning from the mental lexicon (Schettino et al., 2010). Overall, different executive deficits might account for the inefficient comparison between attributes of distant semantic domains (i.e., novel metaphors) and for the retrieval of a stored schema of meaning (as for conventional metaphors). This aspect, however, deserves future attention since only very limited and inconclusive evidence is available so far.

A further concern consists in the role of context processing during metaphor comprehension. Chakrabarty et al. (2014), who systematically controlled for the effect of sentence context, found that contextual information can reduce the misinterpretation of conventional metaphor to a greater extent than novel metaphor. Therefore, processing of conventional metaphor is less severely impaired because it can benefit of the facilitation effect provided by the availability of contextual information (i.e., metaphorical association embedded in a sentence), which in other terms supports the processes of retrieval of the conventional meaning from long term memory (Giora, 2003). Conversely, the comprehension of novel metaphor, whose meaning is not stored into mental lexicon, turns out to be more disrupted. As the authors underline, the facilitation effect due to contextual cues is 37.1% less in the case of novel metaphor comprehension as compared to conventional metaphor. Results of Chakrabarty et al. (2014) in addition, imply that context processing is preserved in schizophrenia. This account is inconsistent with two studies of Iakimova et al. (2005, 2006) that support instead the failure of context processing, both in literal and metaphorical language. Nonetheless, the apparent inconsistency might be the result of the different methods adopted to investigate context processing. First, the latter studies did not compare two-words metaphors with metaphorical sentence (as in Chakrabarty et al., 2014), but all the experimental stimuli were metaphorical sentences (Iakimova et al., 2005, 2006). Moreover, Iakimova et al. (2005) probed the integration of contextual information by displaying sentence words in rapid serial visual presentation, whereas Chakrabarty et al. (2014) presented the whole sentence on the screen; these two types of stimulus presentation may pose a different working memory demand. Second, Iakimova et al. (2005, 2006) were not primarily interested in exploring salience (i.e., their metaphors consisted in commonly used expressions) and this might have somehow prevented to differentiate between literal and metaphoric comprehension in the schizophrenics group. Overall, these studies (Iakimova et al., 2005, 2006; Chakrabarty et al., 2014) provide partially comparable pieces of evidence on context processing during metaphor comprehension in schizophrenia. Future work addressing this issue with respect to the salience of metaphor is thus desirable.

Finally, several studies addressed the role of defective mind-reading in metaphor comprehension in individuals with schizophrenia (Drury et al., 1998; Herold et al., 2002; Langdon et al., 2002; Mo et al., 2008; Champagne-Lavau and Stip, 2010). Among the studies that directly examined the relationship between these two deficits (Langdon et al., 2002; Mo et al., 2008; Champagne-Lavau and Stip, 2010), two out of three statistically accounted for a link (Mo et al., 2008; Champagne-Lavau and Stip, 2010). However, only Champagne-Lavau and Stip (2010) give explicit support to the hypothesis that ToM is a possible antecedent of metaphor misinterpretation, and specifically, of conventional metaphor misinterpretation. Overall, ToM deficit seems to be more likely involved in the impaired comprehension of other figurative expressions that pose a greater demand for mind-reading, e.g., irony (Langdon et al., 2002).

To sum up, metaphor misinterpretation in schizophrenia might be the sign of a disrupted communication between executive functions and semantics. At the least for now, a more detailed outline of the deficit is prevented by the fact that evidence concerning each deficit is very limited, mostly as concerned the executive domain. Moreover, a substantial variety of theoretical backgrounds both in terms of figurative language comprehension models and metaphoric stimulus type critically reduces the possibility for systematic comparisons. Finally, the investigation has been often conducted on relatively small samples, a fact that strongly reduce statistical power of the results.

## Suggestions for Future Directions

The comprehension of metaphor in schizophrenia is still an open issue that warrants future experimental consideration at least for two reasons.

First, metaphors represent a real-world communication problem for schizophrenics. Because metaphors are ubiquitous in ordinary conversation (Lakoff and Johnson, 1980), schizophrenics are very often exposed to misunderstand the speaker’s intention of conveying a metaphoric meaning. This increases patients’ proneness to violate the conversational maxim of cooperation (Grice, 1975) with implications for social functioning (e.g., social withdrawal; Mitchell and Crow, 2005; Champagne-Lavau and Stip, 2010).

Second, neuropsychological and neurofunctional underpinnings of metaphor misinterpretation may intriguingly cast light on the neuropathological organization of the syndrome (Chakrabarty et al., 2014; Faust and Kenett, 2014). Crow (1997) has hypothesized that nuclear symptoms of schizophrenia might be “spill-over effects” of the reduced lateralization of neural areas strictly devoted to the faculty of language and that the observed difficulties with metaphoric speech in schizophrenia might be a side-effect of the aberrant hemispheric organization of the brain (Mitchell and Crow, 2005). Intriguingly, metaphor comprehension relies on fronto-temporal cortices recruitment, the neural areas mostly affected by the neuropathology of schizophrenia (Ellison-Wright et al., 2008; Schultz et al., 2009; Gutiérrez-Galve et al., 2014).

Several aspects deserve future attention. First, the possible relation among metaphoric comprehension impairment, different diagnostic profiles and psychopathology. Second, the assessment of other kinds of metaphors beyond nominal metaphors (e.g., predicate metaphors; Cacciari et al., 2011). A further issue to emphasize is the fact that the examination of schizophrenics’ metaphoric comprehension has been mostly accomplished on patients with well-established illness, ignoring the impact of different antipsychotic drugs on cognitive functions (Ferreira et al., 2016). Conversely, there is very little evidence of concretism at the initial stages of schizophrenia (Anand et al., 1994; Baltaxe and Simmons, 1995; Perlini et al., 2018). Studying metaphoric language impairment in first episode psychoses (FEPs) can take advantage from the fact that the confounding effects of chronicity and antipsychotic medication on cognition and neuroanatomy can be relatively ruled out. Several aspects of the illness, likely related to metaphor misunderstanding, are detectable since the onset, such as language disorders (Ayer et al., 2016; Roche et al., 2016) and the general neurocognitive deterioration (Mesholam-Gately et al., 2009). In the light of this, the investigation of metaphor comprehension at the very beginning of the illness may crucially contribute to the investigation of the neurocognitive mechanisms underlying concretism.

Moreover, the elucidation of the underpinnings of metaphor comprehension in FEPs could take indirectly advantage from the assessment of language outcome following a cognitive training plan. Protocols for cognitive remediation usually target several neurocognitive functions (e.g., Lindenmayer et al., 2008; Vita et al., 2011) and they seem to moderately ameliorate global cognition in schizophrenic patients (McGurk et al., 2007; Wykes et al., 2011) with effects of learning-induced neuroplasticity (Haut et al., 2010; Isaac and Januel, 2015), even in response to untrained tasks (Ramsay and MacDonald, 2015). This raises the possibility that cognitive intervention on FEPs (that generally benefit more than chronic patients from cognitive treatment plans) might positively impact on metaphoric processing, providing indirect information about the cognitive abilities that better account for the initial decline of metaphor comprehension.

## Author Contributions

All authors substantially contribute to the final version of the manuscript. IR wrote the first draft of the manuscript. CP made critical revisions and approved it for publication. PB discussed the initial structure of the paper.

## Conflict of Interest Statement

The authors declare that the research was conducted in the absence of any commercial or financial relationships that could be construed as a potential conflict of interest.
